# Longitudinal associations of light-intensity physical activity with quality of life, functioning and fatigue after colorectal cancer

**DOI:** 10.1007/s11136-020-02566-7

**Published:** 2020-07-02

**Authors:** E. H. van Roekel, J. Duchâteau, M. J. L. Bours, L. van Delden, J. J. L. Breedveld-Peters, J. L. Koole, M. Kenkhuis, P. A. van den Brandt, R. L. Jansen, I. Kant, V. Lima Passos, K. Meijer, S. O. Breukink, M. L. G. Janssen-Heijnen, E. Keulen, M. P. Weijenberg

**Affiliations:** 1 Department of Epidemiology, GROW School for Oncology and Developmental Biology, Maastricht University, P.O. Box 616, 6200 MD Maastricht, The Netherlands; 2 Department of Epidemiology, CAPHRI School for Public Health and Primary Care, Maastricht University, Maastricht, The Netherlands; 3 Department of Medical Oncology, GROW School for Oncology and Developmental Biology, Maastricht University Medical Center, Maastricht, The Netherlands; 4 Department of Methodology and Statistics, Maastricht University, Maastricht, The Netherlands; 5 Department of Human Movement Science, NUTRIM School for Nutrition and Translational Research in Metabolism, Maastricht University, Maastricht, The Netherlands; 6 Department of Surgery, GROW School for Oncology and Developmental Biology, Maastricht University Medical Center+, Maastricht, The Netherlands; 7 Department of Clinical Epidemiology, VieCuri Medical Center, Venlo, The Netherlands; 8 Department of Internal Medicine and Gastroenterology, Zuyderland Medical Centre, Sittard-Geleen, The Netherlands

**Keywords:** Colorectal cancer survivor, Light-intensity physical activity, Longitudinal, Health-related quality of life, Functioning, Fatigue

## Abstract

**Purpose:**

Evidence from cross-sectional studies suggests that higher levels of light-intensity physical activity (LPA) are associated with better health-related quality of life (HRQoL) in colorectal cancer (CRC) survivors. However, these associations have not been investigated in longitudinal studies that provide the opportunity to analyse how within-individual changes in LPA affect HRQoL. We investigated longitudinal associations of LPA with HRQoL outcomes in CRC survivors, from 6 weeks to 2 years post-treatment.

**Methods:**

Data were used of a prospective cohort study among 325 stage I–III CRC survivors (67% men, mean age: 67 years), recruited between 2012 and 2016. Validated questionnaires were used to assess hours/week of LPA (SQUASH) and HRQoL outcomes (EORTC QLQ-C30, Checklist Individual Strength) at 6 weeks, and 6, 12 and 24 months post-treatment. We applied linear mixed regression to analyse longitudinal confounder-adjusted associations of LPA with HRQoL.

**Results:**

We observed statistically significant longitudinal associations between more LPA and better global quality of life and physical, role and social functioning, and less fatigue over time. Intra-individual analysis showed that within-person increases in LPA (per 8 h/week) were related to improved HRQoL, including better global quality of life (*β* = 1.67, 95% CI 0.71; 2.63; total range scale: 0–100) and less fatigue (*β* = − 1.22, 95% CI − 2.37; − 0.07; scale: 20–140). Stratified analyses indicated stronger associations among participants below the median of moderate-to-vigorous physical activity (MVPA) at diagnosis.

**Conclusion:**

Higher levels of LPA were longitudinally associated with better HRQoL and less fatigue in CRC survivors up to two years post-treatment. Further prospective studies using accelerometer data are necessary to inform development of interventions targeting LPA.

**Electronic supplementary material:**

The online version of this article (10.1007/s11136-020-02566-7) contains supplementary material, which is available to authorized users.

## Introduction

Colorectal cancer (CRC) ranks as the third most prevalent cancer in men and second in women worldwide, with an estimated 4.8 million people diagnosed with CRC in the past 5 years [1]. Population ageing is an important cause of an increased incidence of CRC [2]. At the same time, survival rates are steadily increasing because of earlier diagnosis and improvements in treatment, leading to a global increase in the number of CRC survivors. CRC and/or its treatment often lead to long-term complaints such as fatigue and bowel problems [3], which have a negative impact on the health-related quality of life (HRQoL) of CRC survivors [4]. Since the number of CRC survivors is rising, it is of interest to identify factors related to long-term HRQoL outcomes, to provide leads for interventions to improve well-being after CRC.

Physical activity is a modifiable lifestyle behaviour that has been shown in many observational studies to be associated with better HRQoL outcomes in CRC survivors up to 10 years post-treatment [5]. Previous research mostly focused on moderate-to-vigorous physical activity (MVPA), which is the intensity of physical activity that is recommended in current physical activity guidelines for cancer survivors [6–8]. MVPA consists of activities that expend ≥ 3 metabolic equivalent of tasks (METs) and includes for example brisk walking and cycling [9]. More MVPA has been shown to be beneficially associated with physical, role and social functioning and fatigue after CRC in observational studies [5]. Intervention studies in survivors of other cancer types (mostly breast cancer) have shown beneficial effects of exercise interventions (including MVPA) on HRQoL [10]. However, the few randomized controlled trials that studied the efficacy of exercise interventions on HRQoL in CRC survivors found generally no effects [5]. In addition, previous studies found that CRC survivors are often physically inactive and spend around two-thirds of their waking time in sedentary behaviour (i.e. sitting/lying with a low energy expenditure) [11, 12].

An emerging body of evidence points to beneficial effects of light-intensity physical activity (LPA) in elderly individuals [13] and specifically CRC survivors [5]. LPA includes activities with an intensity of < 3 METs, for example light walking or light household activities. Replacing sedentary behaviour by LPA may be a promising additional target for interventions to improve health and well-being among CRC survivors, particularly among those survivors who may not be able or willing to engage in sufficient amounts of MVPA because of their older age and/or present comorbidities [14]. In a previous cross-sectional study from our research group, we found that more self-reported time spent on LPA was associated with better physical and role functioning in 2–10-year post-treatment CRC survivors [15]. These associations remained upon adjustment for MVPA, indicating that the associations of LPA with HRQoL outcomes after CRC are (partly) independent from those of MVPA. We also observed in the same population, using accelerometer data and isotemporal substitution modeling, that substituting sedentary time with standing (a type of LPA) may be beneficially related to HRQoL outcomes including physical functioning and fatigue [11]. Although previous studies in older cancer survivors (mainly breast cancer) [14, 16] and elderly individuals from the general population [13] have found that more LPA was longitudinally associated with better physical function/health and psychological well-being, to our knowledge, no evidence is yet available on longitudinal associations between LPA and HRQoL after CRC.

Altogether, there is a need for more research on longitudinal associations of LPA with HRQoL outcomes in CRC survivors. Insight into potential effects of LPA on HRQoL may provide leads for better tailoring of physical activity interventions and guidelines for CRC survivors. Therefore, this study aimed to investigate how self-reported time spent in LPA was longitudinally associated with several HRQoL outcomes in CRC survivors up to 2 years post-treatment. Specifically, we examined associations with global quality of life, physical, role and social functioning, and fatigue.

## Methods

### Study design and population

**Fig. 1:**
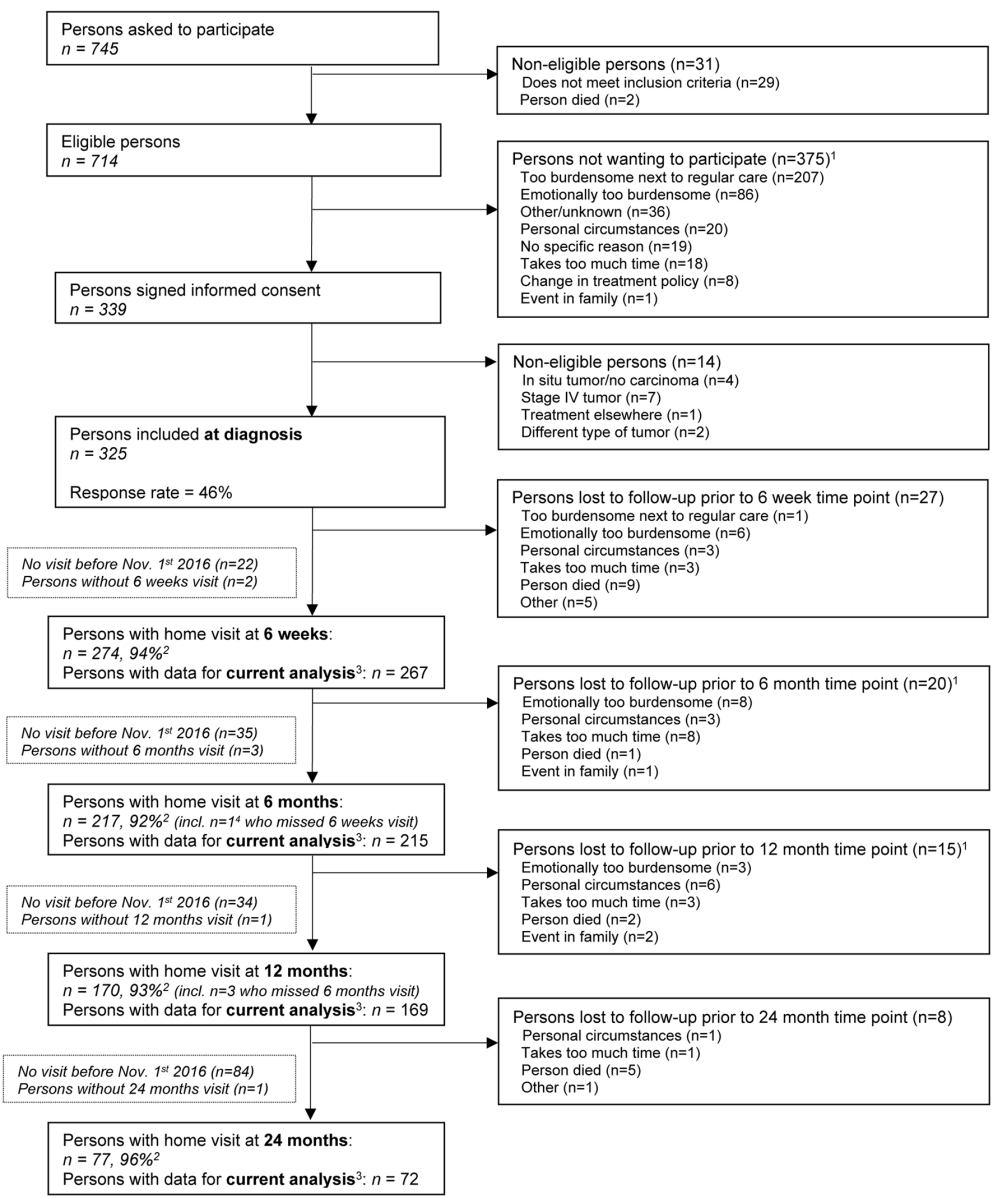
Flow diagram of participants and measurements within the EnCoRe study and the number of post-treatment measurements included in the analyses presented in this manuscript. Data collected up until November 1st 2016 were included in the analyses. ^1^Totals do not add up because some individuals reported multiple reasons for non-participation. ^2^Response rate = (persons with home visits)/(persons with home visits + persons lost to follow-up − persons died). The declining numbers of participants at subsequent time points are predominantly due to the fact that not all participants had reached yet these time points on November 1st 2016. ^3^Since the current study was focused on light physical activity and quality of life after colorectal cancer treatment, only post-treatment measurements with available data on self-reported physical activity, metabolites and covariates were included in the analyses. ^4^Other person who also missed 6 weeks of visit did not have follow-up visit before Nov. 1st 2016

This study analysed data from the Energy for life after ColoRectal cancer (EnCoRe) study, an ongoing prospective cohort study initiated in 2012. Stage I–III CRC patients are recruited at diagnosis from three participating hospitals in the southeastern part of the Netherlands: Maastricht UMC+, VieCuri Medical Center, and Zuyderland Medical Centre [17, 18]. Participants are followed up with repeated measurements at 6 weeks, 6 months, 1 year, 2 years and 5 years after the end of treatment, by trained research dieticians during home visits. For the current study, data of 325 participants who were recruited at diagnosis between April 18th, 2002 and November 1st, 2016 were used (response rate: 46%). Specifically, available post-treatment data on LPA and HRQoL were analysed of *n* = 267 survivors at 6 weeks, *n* = 215 at 6 months, *n* = 169 at 1 year and *n* = 72 at 2 years post-treatment (Fig. 1). Response rates at post-treatment measurements were > 90% and mortality during follow-up was negligible (*n* = 17). The declining numbers of participants with available data at post-treatment follow-up time points and the lack of 5-year post-treatment measurements are (predominantly) due to the fact that not all participants included at diagnosis from April 2012 onwards had reached these time points yet on November 1st, 2016. Inclusion criteria for the EnCoRe study comprised men and women aged minimum 18 years old at diagnosis, living in the Netherlands and able to understand and speak Dutch. Individuals with stage IV CRC and comorbidities obstructing successful study participation, including cognitive disorders such as Alzheimer’s disease or severe vision or hearing disorders, were excluded. The EnCoRe study has been approved by the Medical Ethics Committee of the Academic Hospital Maastricht and Maastricht University, The Netherlands (Netherlands Trial Register no. NL6904). All participants gave written informed consent.

## Data collection and measurements

### Light-intensity physical activity

Self-reported time spent in LPA was measured at each time point with the validated Short QUestionnaire to ASsess Health-enhancing physical activity (SQUASH) [19], which assessed the level of physical activity in the previous week. The frequency (days/week), duration (time/day) and intensity (light, moderate or vigorous) of commuting (walking and cycling), household, work and leisure time activities (walking, cycling, gardening, odd jobs and up to four sports) were assessed. Ainsworth’s Compendium of Physical Activities was used to assign MET values to different activities [9]. Based on the MET value assigned, activities were categorized as either LPA (< 3 MET; e.g. light household or light work activities), or MVPA (≥ 3 MET; e.g. vigorous household work, walking and sports). For each participant, total time spent on LPA and MVPA (hours/week) was estimated by aggregating the time spent in all activities categorized as LPA and MVPA, respectively. The SQUASH has been found to be fairly reliable (Spearman’s *ρ* 0.57–0.58) [19, 20]. Comparable to other physical activity questionnaires, its relative validity in measuring hours/week in different activities appeared limited compared to accelerometer data (Spearman’s *ρ* 0.20 for light-intensity physical activity, *ρ* 0.40 for moderate intensity; and *ρ* 0.35 for vigorous intensity) [20].

### Health-related quality of life outcomes

HRQoL outcomes including global quality of life, and physical, role and social functioning were measured at each time point with the cancer-specific European Organisation for Research and Treatment of Cancer Quality of Life Questionnaire-Core 30 (EORTC QLQ-C30, version 3.0) [21, 22]. Scores (0–100 points) were calculated with higher scores representing better quality of life or functioning. Validation studies found that the EORTC QLQ-C30 has satisfactory test–retest reliability (Pearson’s *r*: 0.58–0.75), internal consistency (Cronbach’s *α* > 0.70), item convergent validity (correlation of item scores with own subscale, Spearman’s *ρ* > 0.40), and discriminant validity (correlation of item scores with own subscale higher than with other subscales) [23, 24]. Furthermore, the subscales were shown to be able to differentiate between individuals with known differences in health status (e.g. newly diagnosed cancer patients and patients under treatment) [23, 24].

Fatigue was measured using the Checklist Individual Strength (CIS), which was initially developed for patients with chronic fatigue syndrome [25] but has also been applied in cancer survivors [26]. The CIS is composed of 20 items of which scores are summed to a total fatigue score (20–140), with higher scores indicating more fatigue. In patients with multiple sclerosis, the test–retest reliability of the CIS was found to be high (intra-class correlation coefficient: 0.81) [27]. In another study in a general population, the CIS score was able to discriminate between individuals with known differences in fatigue levels (healthy individuals, individuals fatigued for somatic reasons and individuals with mental fatigue) [28].

### Other factors

Sociodemographic characteristics including age (years), sex and highest attained education level (low, medium or high) were self-reported by participants at diagnosis. Smoking status (never, former or current) was assessed through self-report at every time point. Clinical characteristics including tumour site (colon/rectum), cancer stage (I, II or III), chemotherapy and radiotherapy (both, yes/no) were collected from clinical records. Body mass index (BMI; kg/m^2^) was calculated at each time point based on measurements of the weight (kg) and height (m) of the participants by trained dieticians. The number of comorbidities (0, 1 or ≥ 2) was assessed using the 13-item Self-Administered Comorbidity Questionnaire, at each post-treatment time point [29].

### Statistical analyses

Linear mixed regression models were used to analyse overall longitudinal associations of LPA with each of the HRQoL outcomes between 6 weeks up to 2 years post-treatment. LPA was modelled continuously per 8 h/week being approximatively half the interquartile range of this variable. First, regular mixed-model analysis was applied to examine the *overall* longitudinal relationship between LPA and each of the HRQoL outcomes, modelled as time-dependent variables. Obtained regression coefficients from this longitudinal analysis are a weighted average of a between-subject component (how differences in LPA *between* individuals on average are related to the outcome) and a within-subject component (how changes in LPA *within* individuals on average are related to the outcome) [30]. To disentangle these between- and within-subject components, we next applied a hybrid modelling method where both of these components were modelled simultaneously [30]. According to this method, the between-subject component was modelled as the mean value of LPA for each individual across time points, while the within-subject component was modelled as the difference of an individual’s LPA level at each time point with the mean value of LPA of that individual across time points (deviation score) [30]. The obtained regression coefficients will be referred to as inter-individual associations (i.e. between-subject component) and intra-individual associations (i.e. within-subject component), respectively. We adjusted regression models for a priori defined confounders using a stepwise approach. The first model included the main covariates age (years), sex and time since end of treatment (months). In a second model, we further adjusted for additional potential confounders identified based on previous literature [15], including number of comorbidities (0, 1 or ≥ 2), BMI (kg m^2^), chemotherapy (yes/no), cancer stage (I, II or III) and MVPA (hours/week). Finally, we applied the 10% change-in-estimate method [31] with forward selection to identify additional potential confounders, including radiotherapy (yes/no), cancer site (colon/rectum), education level (low/middle/high), current smoking status (never/former/current), having a partner (yes/no), LPA at diagnosis (hours/week) and BMI at diagnosis (kg/m^2^). This final step resulted in LPA at diagnosis being included as a covariate in the analysis of role functioning. We tested whether adding a random slope for LPA improved the model fit using the likelihood ratio test; this test was non-statistically significant for all models indicating that a random slope was not necessary. Since LPA was not normally distributed (i.e. skewed to the right), we also conducted sensitivity analysis using natural log-transformed LPA as the independent variable to check if results were similar to those from our main analysis using untransformed LPA.

To examine potential interaction between LPA and MVPA, sex, age and chemotherapy treatment, we included product-terms for LPA with each of these variables in separate models. In addition, we performed stratified analyses (using the median value at diagnosis as cut-off for MVPA and the mean value at diagnosis as cut-off for age), to describe associations of LPA with the HRQoL outcomes in subgroups. We also investigated the effect of time since treatment on the association of LPA and HRQoL by adding an interaction term between LPA and time since the end of treatment, and conducted these analyses separately for time modelled continuously (to investigate linear interaction effects) as well as for the separate post-treatment time points (to explore potential non-linear interaction). To explore the temporal direction of associations, time-lag analyses were performed in which longitudinal associations of LPA with HRQoL outcomes at a later time point were modelled [32].

Analyses were performed with Stata (version 15), and *p* values less than 0.05 (two-sided tests) were considered statistically significant.

## Results

### Participant characteristics

Participants (*n* = 325) were on average 67 years old (SD: 9.5) at diagnosis (Table 1) and approximately two-thirds were men (67.1%). At diagnosis, 174 participants (53.5%) were former smokers, 101 (31.1%) were never smokers and 42 (12.9%) reported current smoking (data missing for eight participants). The sample comprised 86 normal weight (26.5%, BMI 18.5–24.4 kg/m^2^), 136 overweight (41.8%, BMI 25–29.9 kg/m^2^), 102 obese individuals (31.4%, BMI ≥ 30 kg/m^2^) and one individual with underweight (BMI < 18.5 kg/m^2^) at diagnosis. A total of 198 participants (60.9%) were diagnosed with colon cancer and 127 with rectal cancer (39.1%). Almost half of the participants were diagnosed with stage III CRC (46.8%), and 27.4% and 22.2% with stage I and stage II disease, respectively. A total of 137 participants (42.2%) received treatment with (neo-)adjuvant chemotherapy, 89 (27.4%) were treated with radiotherapy and 68 (20.9%) received both chemo- and radiotherapy. At 6 weeks post-treatment, almost half of the study participants reported to have two or more comorbidities (45.5%) such as hypertension and osteoarthritis.

**Table 1:** Demographic, lifestyle and clinical characteristics of participants at colorectal cancer diagnosis (*n* = 325)

Men, *n* (%)	218 (67.1)
Age (years), mean (SD)	66.5 (9.5)
Smoking status^a^, *n* (%)	
Never	101 (31.1)
Former	174 (53.5)
Current	42 (12.9)
Educational level^a^, *n* (%)	
Low	90 (27.7)
Medium	122 (37.5)
High	105 (32.3)
Treatment centre, *n* (%)	
Maastricht UMC+	200 (61.5)
VieCuri Medical Center	86 (26.5)
Zuyderland Medical Centre	39 (12)
BMI categories (kg/m^2^), *n* (%)	
Underweight (BMI < 18.5)	1 (0.0)
Normal weight (BMI 18.5–24.9)	86 (26.5)
Overweight (BMI 25–29.9)	136 (41.8)
Obese (BMI > 30)	102 (31.4)
Cancer type, *n* (%)	
Colon	198 (60.9)
Rectum	127 (39.1)
Cancer stage ^b^, *n* (%)	
I	89 (27.4)
II	72 (22.2)
III	152 (46.8)
Received radiotherapy, *n* (%)	89 (27.4)
Received chemotherapy, *n* (%)	137 (42.2)

*BMI* body mass index, *Maastricht UMC*+Maastricht University Medical Centre+

^a^Data on smoking status and educational level were missing for 8 participants for both variables (2.5%)

^b^Data on cancer stage were missing for 12 participants (3.7%)

**Fig. 2:**
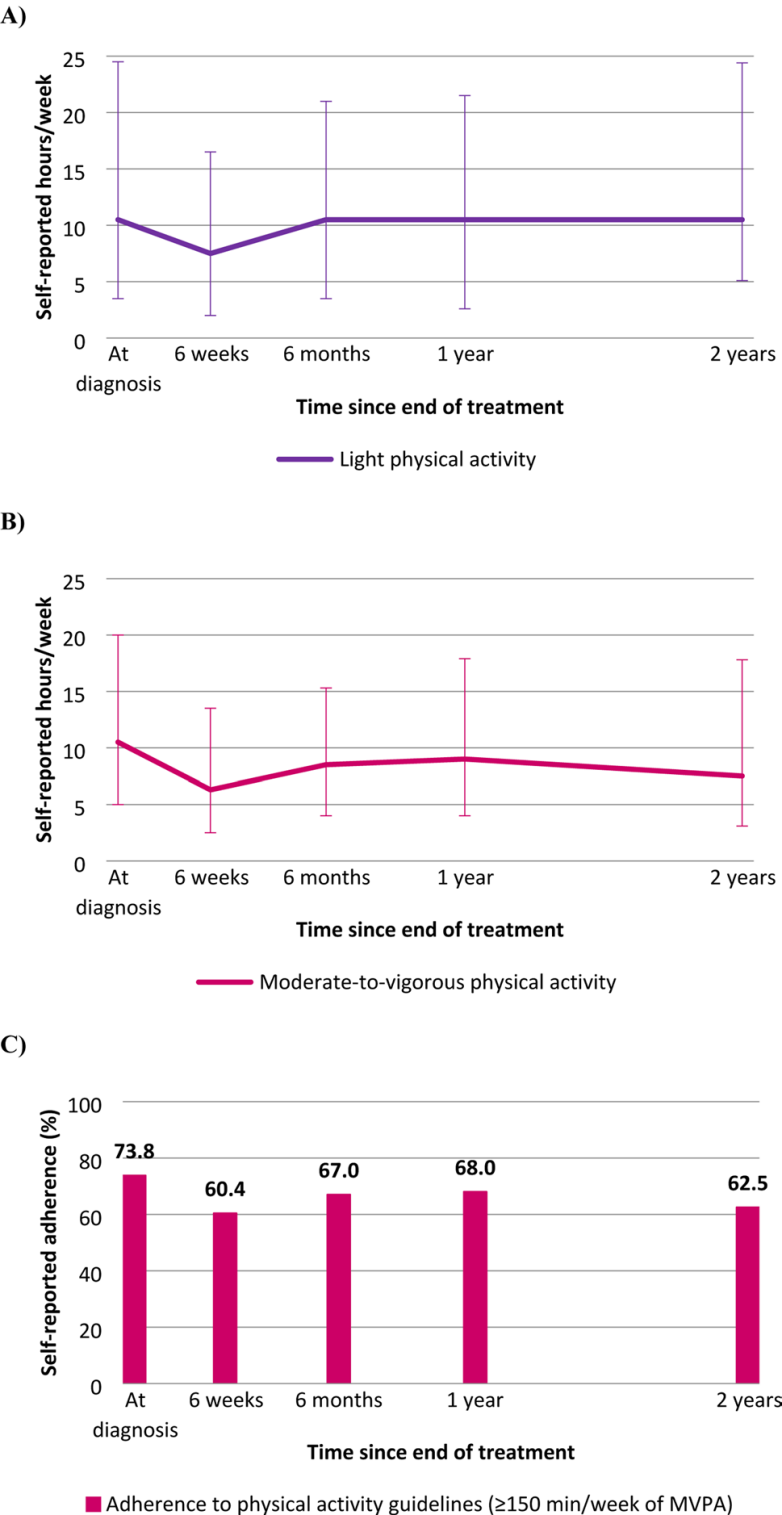
Medians and interquartile ranges of self-reported **a** hours/week of light-intensity physical activity (LPA) and **b** moderate-to-vigorous physical activity (MVPA), and **c** percentage self-reported adherence to Dutch physical activity guidelines (≥ 150 min/week of MVPA) at diagnosis and at post-treatment time points among colorectal cancer survivors included in the current analysis

The median self-reported time spent on LPA and MVPA at diagnosis was 10.5 h/week for both variables (interquartile ranges 3.3–21.6 and 5.0–19.6, respectively; Fig. 2). A decline in median reported time spent in LPA was observed from diagnosis to the 6-week post-treatment measurement (median, 7.5 h/week), and the median increased afterwards to levels similar to those observed at diagnosis. At all time points, the majority of self-reported time spent on LPA consisted of light household work. In total, 234 participants (73.8%) reported to adhere to the Dutch physical activity guidelines at diagnosis (i.e. at least 150 min/week of MVPA), whereas at 6 weeks, 60.4% adhered to the guidelines and this percentage increased to 68% at 12 months and 62.5% at 24 months. Spearman correlation coefficients between LPA measured across different time points ranged from 0.56 to 0.78, indicating moderate to strong longitudinal correlations. Spearman correlation coefficients between hours/week of LPA and MVPA at the different time points were weak and mostly negative, ranging from -0.26 to 0.05.

Mean scores of global quality of life and functioning outcomes increased from the 6-week to the 24-month post-treatment time point, and mean fatigue scores decreased over time (Table 2). Spearman correlation coefficients indicated weak to strong correlations of each HRQoL outcome across time points (range 0.16–0.88), with slightly stronger correlations at later time points. Across HRQoL outcomes, moderate correlations were observed at 6 weeks (range 0.43 to 0.63 among quality of life and functioning outcomes; and − 0.64 to − 0.51 for fatigue with the other outcomes), and similar correlations were observed at other time points.

**Table 2:** Quality of life, functioning and fatigue outcomes at post-treatment measurements of included participants

	Post-treatment measurements			
	6 weeks (*n* = 267)	6 months (*n* = 215)	12 weeks (*n* = 169)	24 months (*n* = 72)
Global quality of life (0–100)^a^, mean (SD)	74.1 (18.6)	76.8 (19.1)	77.5 (18.2)	79.6(18.5)
Physical functioning (0–100)^a^, mean (SD)	76.5 (19.6)	81.9 (18.6)	82.6 (19.5)	84.0 (19.2)
Role functioning (0–100)^a^, mean (SD)	70.5 (28.1)	80.5 (24.3)	84.0 (26.5)	88.0 (19.0)
Social functioning (0–100)^a^, mean (SD)	81.5 (22.2)	89.2 (19.4)	90.5 (18.5)	94.2 (12.6)
Fatigue (20–140)^a,b^, mean (SD)	62.9 (26.6)	59.2 (27.4)	54.4 (26.3)	51.5 (25.2)

^a^Higher scores indicate better global quality of life, physical, role and social functioning and more fatigue

^b^Data on fatigue were missing for 3 participants at 6 weeks, 2 participants at 6 months and 1 participant at 12 months

### Longitudinal associations of light-intensity physical activity with quality of life outcomes

In fully adjusted models assessing overall longitudinal associations (Table 3), time spent on LPA (per 8 h/week) was statistically significantly associated with all investigated outcomes. Specifically, we observed that more LPA was related to better global quality of life (*β* = 1.35, 95% CI 0.55; 2.15), physical functioning (*β* = 1.17, 95% CI 0.54; 1.79), role functioning (*β* = 1.98, 95% CI 0.69;3 .26) and social functioning (*β* = 1.22, 95% CI 0.31; 2.13), and to lower fatigue (*β* = − 1.3, 95% CI − 2.31; − 0.29). When applying hybrid modelling to disentangle intra- and inter-individual associations of LPA with HRQoL outcomes, we observed both intra- and inter-individual associations with similar directions of associations and mostly a similar magnitude of associations as in the overall analysis (Table 3). In particular, results from the intra-individual analysis indicated that an increase in LPA over time within individuals (per 8 h/week) was significantly associated with better global quality of life (*β* = 1.67, 95% CI 0.71; 2.63), physical functioning (*β* = 1.14, 95% CI 0.43; 1.84) and role functioning (*β* = 1.98, 95% CI 0.47;3.49), and with less fatigue (*β* = − 1.22, 95% CI − 2.37; − 0.07). We also observed that an increase in LPA over time was related to better social functioning, although the association was not statistically significant (*β* = 1.09, 95% CI − 0.04; 2.22). Generally, results of inter-individual analyses indicated similar direction and size of associations based on regression coefficients, although none were statistically significant. Results from the fully adjusted model were similar although slightly attenuated when compared to the model adjusted only for sex, age and time since the end of treatment. Results of the analysis of ln-transformed LPA were similar to those obtained in the main analysis (Supplemental Table 1).

**Table 3:** Longitudinal associations of light-intensity physical activity (per 8 h/week) with quality of life, functioning and fatigue outcomes in colorectal cancer survivors, including confounder-adjusted overall and intra- and inter-individual associations

	Global quality of life (0–100)		Physical functioning (0–100)		Role functioning (0–100)		Social functioning (0–100)		Fatigue (20–140)	
	*β*	95% CI	*β*	95% CI	*β*	95% CI	*β*	95% CI	*β*	95% CI
*Model I*^a^										
Overall association^b^	1.55	0.74; 2.36	1.38	0.74; 2.02	1.89	0.71; 3.04	1.32	0.42; 2.22	− 1.67	− 2.70; − 0.64
Intra-individual^c^	1.88	0.92; 2.85	1.38	0.67; 2.08	2.32	0.81; 3.82	1.29	0.18; 2.41	− 1.59	− 2.75; − 0.43
Inter-individual^d^	0.78	− 0.67; 2.23	1.39	− 0.09; 2.87	1.19	− 0.69; 3.09	1.35	− 0.18; 2.87	− 1.95	− 4.16; 0.26
*Model II*^e^										
Overall association^c^	1.35	0.55; 2.15	1.17	0.54; 1.79	1.98	0.69; 3.26	1.22	0.31; 2.13	− 1.30	− 2.31; − 0.29
Intra-individual^d^	1.67	0.71; 2.63	1.14	0.43; 1.84	1.98	0.47; 3.49	1.09	− 0.04; 2.22	− 1.22	− 2.37; − 0.07
Inter-individual^e^	0.62	− 0.80; 2.04	1.23	− 0.16; 2.61	1.95	− 0.46; 4.36	1.43	− 0.10; 2.96	− 1.53	− 3.65; 0.59

*β* beta-coefficient, *CI* confidence interval

^a^Model adjusted for sex (male/female), age (years) and time since the end of treatment (months)

^b^The beta-coefficients represent the overall longitudinal difference in the outcome score per 8 h/week difference in light-intensity physical activity, including intra- and inter-individual associations. Higher outcome scores indicate better global quality of life, physical, role and social functioning and more fatigue

^c^The beta-coefficients represent the change in the outcome score over time within individuals per 8 h/week increase in light-intensity physical activity

^d^The beta-coefficients represent the difference in the outcome score between individuals, per 8 h/week difference in light-intensity physical activity

^e^Model adjusted for sex (male/female), age (years) and time since the end of treatment (months), chemotherapy (yes/no), number of comorbidities (0, 1 or ≥ 2), body mass index (kg/m^2^) cancer stage (I, II or III) and self-reported moderate-to-vigorous intensity physical activity (hours/week). For role functioning, additional adjustment was performed for light-intensity physical activity at diagnosis (hours/week) (see Methods)

### Stratified analyses

Statistically significant interactions between LPA and MVPA were found for all HRQoL outcomes (Table 4), and between LPA and age for physical, role and social functioning (Supplemental Table 2). Stratified analyses showed overall, intra- and inter-individual associations among individuals spending < 10.5 h/week in MVPA at diagnosis, while among participants spending ≥ 10.5 h/week mostly very small and non-statistically significant associations were found. Further, stratified analyses indicated stronger overall and intra-individual associations of LPA with HRQoL outcomes among younger vs. older individuals (≤ 67 years vs. > 67 years of age), while inter-individual analyses showed mostly weaker effects in younger participants. We also observed an interaction between LPA and time since end of treatment for fatigue, for both time modelled continuously and at 24 months compared to 6 weeks as reference (Supplemental Table 3), indicating stronger associations of LPA with fatigue over time. In time-lag analyses investigating longitudinal associations between LPA and HRQoL at subsequent time points, we observed non-significant and weaker associations compared to the main analysis (Supplemental Table 4).

**Table 4:** Stratified analyses by MVPA at diagnosis (median cut-off) including overall, intra-and inter-individual longitudinal associations of light-intensity physical activity (per 8 h/week) with quality of life, functioning and fatigue in colorectal cancer survivors

	MVPA < 10.5 h/week (*n* = 111)		MVPA ≥ 10.5 h/week (*n* = 157)		
	*β*	95% CI	*β*	95% CI	P_interaction_^a^
Global quality of life (0–100)					
Overall^b^	1.59	0.56; 2.66	0.04	− 1.22; 1.29	0.164
Intra-individual^c^	1.82	0.54; 3.09	0.31	− 1.41; 2.04	0.016*
Inter-individual^d^	1.17	− 0.59; 2.93	− 0.31	− 2.24; 1.63	0.442
Physical functioning (0–100)					
Overall^b^	1.47	0.64; 2.30	− 0.06	− 0.99; 0.87	0.010*
Intra-individual^c^	1.33	0.36; 2.30	0.09	− 0.96; 1.15	0.017*
Inter-individual^d^	1.86	0.26; 3.46	− 0.65	− 2.72; 1.42	0.422
Role functioning (0–100)					
Overall^b^	2.75	1.12; 4.38	− 0.06	− 2.06; 1.94	0.004*
Intra-individual^c^	2.69	0.74; 4.65	− 0.07	− 2.54; 2.41	0.001*
Inter-individual^d^	2.96	− 0.06; 5.97	− 0.04	− 3.49; 3.42	0.716
Social functioning (0–100)					
Overall^b^	2.13	0.93; 3.32	− 0.78	− 2.13; 0.58	0.015*
Intra-individual^c^	1.93	0.39; 3.46	− 1.04	− 2.81; 0.73	0.034*
Inter-individual^d^	2.44	0.50; 4.38	− 0.45	− 2.67; 1.76	0.292
Fatigue (20–140)					
Overall^b^	− 2.11	− 3.34; − 0.88	1.05	− 0.84; 2.95	0.005*
Intra-individual^c^	− 2.05	− 3.47; − 0.63	1.43	− 0.96; 3.82	0.000*
Inter-individual^d^	− 2.33	− 4.84. 0.18	0.46	− 2.81; 3.73	0.407

Models were adjusted for sex (male/female), age (years) and time since the end of treatment (months), chemotherapy (yes/no), number of comorbidities (0, 1 or ≥ 2), body mass index (kg/m^2^), cancer stage (I, II or III) and self-reported moderate-to-vigorous intensity physical activity (hours/week). For role functioning, additional adjustment was performed for light-intensity physical activity at diagnosis (hours/week) (see Methods)

*β* beta-coefficient, *MVPA* moderate-to-vigorous physical activity, *CI* confidence interval

^a^Statistical interaction was tested by including a product term of light-intensity physical activity with MVPA (< 10.5 versus ≥ 10.5 h/week at diagnosis). Statistically significant interactions (*p* values < 0.05) are denoted with an asterisk (*)

^b^The beta-coefficients represent the overall longitudinal difference in the outcome score per 8 h/week difference in light-intensity physical activity, including intra- and inter-individual associations. Higher outcome scores indicate better global quality of life, physical, role and social functioning and more fatigue

^c^The beta-coefficients represent the change in the outcome score over time within individuals per 8 h/week increase in light-intensity physical activity

^d^The beta-coefficients represent the difference in the outcome score between individuals, per 8 h/week difference in light-intensity physical activity

## Discussion

Within this prospective cohort study, we found that higher levels of LPA (per 8 h/week) were longitudinally associated with better global quality of life and better physical, role and social functioning and less fatigue among CRC survivors. We observed intra-individual associations indicating that an increase in LPA within individuals over time was associated with better HRQoL and functioning and with less fatigue. In addition, we obtained similar results for inter-individual associations, indicating that CRC survivors with higher mean levels of LPA in the first 2 years after treatment reported generally better HRQoL and functioning and less fatigue than CRC survivors who reported a lower average LPA. Stratified analyses indicated stronger associations of LPA with HRQoL in individuals with lower MVPA levels at diagnosis and in younger individuals.

These results are consistent with previous findings. Our research group previously reported that more self-reported LPA was cross-sectionally associated with better physical and role functioning and less disability among CRC survivors who were 2 to 10 years post-diagnosis [15], and that substituting sedentary time with standing (a type of LPA) may be associated with better physical functioning and lower disability and fatigue in this population [11]. In addition, an American study also found cross-sectional and longitudinal associations of more self-reported LPA with better physical functioning in a mixed group of cancer survivors, including CRC survivors [14], whereas another American study observed that more LPA was associated with better mental quality of life outcomes in older female cancer survivors (including CRC) [16]. Further, a cross-sectional study in a sample of healthy older US adults found that more accelerometer-assessed LPA was related to better physical health and well-being [13].

These findings are encouraging in terms of LPA as a potential target for lifestyle interventions to improve health and well-being in CRC survivors. Nevertheless, the regression coefficients we observed were mostly small compared to published guidelines for minimal important changes (MICs) for EORTC QLQ-C30 scores [33] and 0.5 times the SD of the CIS score (recommended in case there is no published MIC available [34]). In addition, results from time-lag analyses suggested that associations between LPA and HRQoL over time may be reciprocal. For example, it could be that participants who experienced fatigue decreased their activity levels, possibly leading to a downward spiral of both increasing fatigue and decreasing LPA. Nevertheless, interventions targeting LPA might break this spiral and thus improve HRQoL in CRC survivors [10]. Intervention studies will be necessary to determine whether changes in LPA may lead to relevant changes in HRQoL outcomes after CRC.

We found interactions between LPA and MVPA for all HRQoL outcomes and for LPA with age for physical, role and social functioning. Stratified analyses showed that LPA was more strongly associated with HRQoL outcomes among individuals with lower MVPA levels. We also observed weak and inverse correlations between LPA and MVPA, and associations of LPA remained significant upon adjustment for MVPA. These results indicate that LPA may be more relevant for HRQoL in individuals with lower MVPA, and that the associations of LPA with HRQoL are likely separate from those of MVPA with HRQoL. Further, we found stronger intra-individual associations of LPA with HRQoL outcomes among younger individuals (≤ 67 years of age). The difference in associations is not likely due to differences in MVPA between younger and older participants, since median levels of MVPA at diagnosis and during follow-up were similar. A potential explanation for this finding is that younger individuals reported higher LPA and larger intra-individual differences in LPA over time in our study. We also found the association between LPA and fatigue being stronger at longer time since treatment. This observation may be due to fatigue being mostly influenced by treatment factors in the first 6 months after treatment and potentially more by other factors including LPA afterwards. The results of these exploratory analyses require further replication in larger samples.

An important strength of this study is its prospective nature with four repeated measurements over time. Other strengths of our study include the high response rates during follow-up (> 90%), comprehensive measurements of relevant aspects of HRQoL and potential confounders and effect modifiers, and the limited number of missing data resulting from intensive data collection methods. Although we cannot rule out the possibility of residual confounding, we were able to adjust our analyses for a comprehensive set of potential confounders including factors related to nutritional and performance status (e.g. BMI, number of comorbidities, time since treatment) which likely influence both physical activity and fatigue in this population. Nevertheless, based on these observational data, we cannot be sure of the direction of associations, and intervention studies will be necessary to infer causality. Another limitation of our study is that selection bias might have occurred since study participants were likely younger and reported a higher level of physical activity and a better HRQoL than the total population of CRC survivors. Indeed, our sample had a mean age at diagnosis of 66.5 years which is lower than the average of 70 years in developed countries [35]. We observed stronger associations with some outcomes in younger individuals, and the overrepresentation of younger CRC survivors may have led to overestimation of associations in our study. We do not expect bias due to selective loss to follow-up in our cohort, since the predominant reason for a lack of follow-up measurements was that participants had not yet reached these time points. Moreover, follow-up response rates were high and mortality was negligible. Importantly, we used linear mixed-model analysis which is a longitudinal data analysis technique that makes use of all available data, including data of participants who had not completed all repeated measurements. Our results indicate that we had sufficient power to detect statistically significant longitudinal associations. In addition, the use of a self-report questionnaire to assess LPA and MVPA may be susceptible to recall error. Further, the main type of LPA assessed by the SQUASH is light household work and light work, whereas other LPA activities such as standing or light walking during leisure time are not included. The incomplete coverage of all aspects of LPA may have resulted in the relatively low level of LPA that we observed in our study (median 10.5 h/week at diagnosis), compared to those observed in a previous study in colon cancer survivors where accelerometers were applied to objectively measure LPA (mean ~ 36 h/week) [12]. Therefore, future studies should preferably use an objective method such as accelerometers to assess LPA and more comprehensive questionnaires to better assess the full spectrum of light-intensity activities.

In conclusion, this longitudinal study confirms earlier findings that spending more time in LPA is longitudinally associated with better global quality of life, physical, role and social functioning, and with less fatigue in CRC survivors up to two years post-treatment. Importantly, we observed that individuals who increased their LPA levels over time reported improved quality of life outcomes and longitudinal associations were more pronounced among participants with lower MVPA levels at diagnosis. Replacing sedentary behaviour by LPA (e.g. household activities, light walking) may be an important target for lifestyle interventions for CRC survivors since many of these individuals are not able to exercise at moderate-to-high-intensity due to their older age and/or comorbidities. Future longitudinal studies with objective accelerometer data are necessary to further investigate how sedentary behaviour and LPA are related to HRQoL, and intervention studies will be necessary to determine the direction of associations between LPA and HRQoL outcomes over time. In addition, qualitative data will need to be collected with regards to preferences of CRC survivors and feasibility of LPA interventions. These results will provide further leads for development of targeted lifestyle interventions to improve the health and well-being of CRC survivors.

## Electronic supplementary material

Below is the link to the electronic supplementary material.

Supplementary file1 (DOCX 56 kb)

## Data Availability

The data supporting the results reported in this article are securely stored at Maastricht University. The corresponding author can be contacted to request access to the data.

## Code availability

The corresponding author can be contacted to request a copy of the scripts used to generate the results included in this manuscript.
