# Supplementary material for: Longitudinal associations of light-intensity physical activity with quality of life, functioning and fatigue after colorectal cancer
Source: Qual Life Res. 2020 Jul 2;29(11):2987–98. doi: 10.1007/s11136-020-02566-7 (PMC7591443; doi:10.1007/s11136-020-02566-7)
Supplement: Supplementary file 1 — Supplementary file1 (DOCX 56 kb) [file 11136_2020_2566_MOESM1_ESM.docx]

| **Supplemental Table 1.** Results from sensitivity analysis of confounder-adjusted associations of natural logarithm-transformed light-intensity physical activity (per 8 hours∙week^-1^ increase) with quality of life, functioning and fatigue in colorectal cancer survivors^a^. | | |
| --- | --- | --- |
|  | β ^b^ | 95% CI |
| Global quality of life (0-100) | 1.55 | 0.33; 2.77 |
| Physical functioning (0-100) | 2.00 | 1.04; 2.96 |
| Role functioning (0-100) | 3.00 | 1.11; 4.94 |
| Social functioning (0-100) | 1.39 | 0.01; 2.78 |
| Fatigue (20-140) | -1.54 | -3.10; 0.02 |
| Abbreviations: β, beta-coefficient; CI, confidence interval.  ^a^ Models including the ln-transformed light-intensity physical activity variable were adjusted for the same covariates as in the main analysis, i.e. sex (male/female), age (years) and time since the end of treatment (months), chemotherapy (yes/no), number of comorbidities (0, 1 or ≥2), body mass index (kg∙m^-^²), cancer stage (I, II or III) and self-reported moderate-to-vigorous intensity physical activity (hours∙week^-1^). For role functioning, additional adjustment was performed for light-intensity physical activity at diagnosis (hours∙week^-1^) (see Methods).  ^b^ The beta-coefficients represent the overall longitudinal difference in the outcome score per 1 unit increase in ln-transformed light-intensity physical activity, which equals the mathematical constant *e* times more light-intensity physical activity in hours∙week^-1^. Higher outcome scores indicate better global quality of life, physical, role and social functioning and more fatigue. | | |

| **Supplemental Table 2.** Stratified analyses by age, sex and chemotherapy treatment including overall, intra-and inter-individual longitudinal associations of light-intensity physical activity (per 8 hours∙week^-1^) with quality of life, functioning and fatigue in colorectal cancer survivors^a^. | | | | | | | | | |
| --- | --- | --- | --- | --- | --- | --- | --- | --- | --- |
|  |  | Age ≤67 years (n=131) | | Age >67 years (n=136) | | |  | | |
|  |  | β | 95% CI | β | 95% CI | | P_interaction_^b^ | | |
| Global quality of life  (0-100) | Overall^c^ | 1.39 | 0.38; 2.41 | 0.78 | -0.53; 2.09 | | 0.217 | | |
|  | Intra-individual^d^ | 1.94 | 0.65; 3.23 | 0.77 | -0.71; 2.24 | | 0.113 | | |
|  | Inter-individual^e^ | 0.51 | -1.11; 2.13 | 0.75 | -1.95; 3.45 | | 0.752 | | |
| Physical functioning  (0-100) | Overall^c^ | 1.52 | 0.78; 2.27 | 0.33 | -0.72; 1.38 | | 0.045* | | |
|  | Intra-individual^d^ | 1.54 | 0.62; 2.45 | 0.01 | -1.10; 1.11 | | 0.018* | | |
|  | Inter-individual^e^ | 1.48 | 0.20; 2.76 | 2.77 | -0.25; 5.79 | | 0.698 | | |
| Role functioning  (0-100) | Overall^c^ | 2.38 | 0.74; 4.01 | 0.63 | -1.41; 2.72 | | 0.172 | | |
|  | Intra-individual^d^ | 2.91 | 0.91; 4.92 | -0.35 | -2.68; 1.98 | | 0.018* | | |
|  | Inter-individual^e^ | 1.27 | -1.55; 4.09 | 4.30 | -0.09; 8.70 | | 0.434 | | |
| Social functioning  (0-100) | Overall^c^ | 1.90 | 0.72; 3.08 | -0.65 | -2.07; 0.78 | | 0.001* | | |
|  | Intra-individual^d^ | 2.05 | 0.50; 3.59 | -0.82 | -2.49; 0.85 | | 0.006* | | |
|  | Inter-individual^e^ | 1.67 | -0.16; 3.49 | -0.17 | -2.79; 2.45 | | 0.071 | | |
| Fatigue  (20-140) | Overall^c^ | -1.25 | -2.54; 0.05 | -0.59 | -2.22; 1.03 | | 0.419 | | |
|  | Intra-individual^d^ | -1.17 | -2.70; 0.35 | -0.28 | -2.04; 1.49 | | 0.247 | | |
|  | Inter-individual^e^ | -1.42 | -3.86; 1.01 | -2.08 | -5.96; 1.79 | | 0.664 | | |
|  |  | Females (n=86) | | Males (n=181) | | |  | | |
|  |  | β | 95% CI | β | 95% CI | | P_interaction_^b^ | | |
| Global quality of life  (0-100) | Overall^c^ | 1.50 | -0.05; 3.05 | 1.28 | 0.33; 2.22 | | 0.540 | | |
|  | Intra-individual^d^ | 2.30 | 0.29; 4.31 | 1.52 | 0.41; 2.63 | | 0.631 | | |
|  | Inter-individual^e^ | 0.37 | -2.04; 2.78 | 0.61 | -1.15; 2.37 | | 0.575 | | |
| Physical functioning  (0-100) | Overall^c^ | 1.86 | 0.54; 3.17 | 0.89 | 0.18; 1.62 | | 0.075 | | |
|  | Intra-individual^d^ | 2.00 | 0.49; 3.52 | 0.88 | 0.08; 1.68 | | 0.202 | | |
|  | Inter-individual^e^ | 1.41 | -1.19; 4.00 | 0.94 | -0.69; 2.57 | | 0.198 | | |
| Role functioning  (0-100) | Overall^c^ | 0.63 | -1.89; 3.16 | 2.40 | 0.90; 3.89 | | 0.882 | | |
|  | Intra-individual^d^ | 1.33 | -1.79; 4.45 | 2.22 | 0.46; 3.97 | | 0.539 | | |
|  | Inter-individual^e^ | -0.67 | -4.97; 3.63 | 2.88 | -0.00; 5.77 | | 0.424 | | |
| Social functioning  (0-100) | Overall^c^ | 0.96 | -0.81; 2.72 | 1.33 | 0.26; 2.41 | | 0.789 | | |
|  | Intra-individual^d^ | 1.27 | -1.13; 3.67 | 1.11 | -0.18; 2.41 | | 0.999 | | |
|  | Inter-individual^e^ | 0.59 | -2.00; 3.19 | 1.77 | -0.14; 3.67 | | 0.733 | | |
| Fatigue  (20-140) | Overall^c^ | -1.78 | -3.92; 0.35 | -0.97 | -2.13; 0.19 | | 0.357 | | |
|  | Intra-individual^d^ | -2.30 | -4.81; 0.20 | -0.86 | -2.16; 0.44 | | 0.202 | | |
|  | Inter-individual^e^ | -0.45 | -4.46; 3.56 | -1.34 | -3.84; 1.16 | | 0.795 | | |
|  |  | Chemotherapy (n=102) | | No chemotherapy (n=165) | |  | |  |  |
|  |  | β | 95% CI | β | 95% CI | | P_interaction_^b^ | |  |
| Global quality of life  (0-100) | Overall^c^ | 1.87 | 0.66; 3.09 | 0.80 | -0.26; 1.87 | | 0.192 | |  |
|  | Intra-individual^d^ | 2.26 | 0.77; 3.56 | 1.17 | -0.10; 2.45 | | 0.242 | |  |
|  | Inter-individual^e^ | 1.31 | -1.00; 3.26 | -0.04 | -1.93; 1.86 | | 0.422 | |  |
| Physical functioning  (0-100) | Overall^c^ | 1.57 | 0.59; 2.55 | 0.84 | 0.03; 1.65 | | 0.575 | |  |
|  | Intra-individual^d^ | 1.44 | 0.31; 2.56 | 0.87 | -0.03; 1.78 | | 0.433 | |  |
|  | Inter-individual^e^ | 2.05 | -0.01; 4.11 | 0.68 | -1.14; 2.49 | | 0.842 | |  |
| Role functioning  (0-100) | Overall^c^ | 2.42 | 0.46; 4.38 | 1.63 | -0.06; 3.31 | | 0.929 | |  |
|  | Intra-individual^d^ | 2.63 | 0.25; 5.00 | 1.40 | -0.59; 3.39 | | 0.371 | |  |
|  | Inter-individual^e^ | 2.07 | -1.50; 5.64 | 2.19 | -0.98; 5.37 | | 0.441 | |  |
| Social functioning  (0-100) | Overall^c^ | 1.74 | 0.36; 3.12 | 0.92 | -0.26; 2.10 | | 0.603 | |  |
|  | Intra-individual^d^ | 1.55 | -0.44; 3.55 | 0.86 | -0.52; 2.23 | | 0.557 | |  |
|  | Inter-individual^e^ | 1.90 | -0.01; 3.81 | 1.07 | -1.19; 3.33 | | 0.927 | |  |
| Fatigue  (20-140) | Overall^c^ | -1.39 | -2.90; 0.11 | -1.18 | -2.55; 0.18 | | 0.966 | |  |
|  | Intra-individual^d^ | -1.16 | -2.91; 0.59 | -1.24 | -2.78; 0.30 | | 0.943 | |  |
|  | Inter-individual^e^ | -2.13 | -5.16; 0.89 | -0.96 | -3.88; 1.96 | | 0.994 | |  |
| Abbreviations: β, beta-coefficient; MVPA, moderate-to-vigorous physical activity; CI, confidence interval.  ^a^ Models were adjusted for sex (male/female), age (years) and time since the end of treatment (months), chemotherapy (yes/no), number of comorbidities (0, 1 or ≥2), body mass index (kg∙m^-^²), cancer stage (I, II or III) and self-reported moderate-to-vigorous intensity physical activity (hours∙week^-1^). For role functioning, additional adjustment was performed for light-intensity physical activity at diagnosis (hours∙week^-1^) (see Methods).  ^b^ Statistical interaction was tested by including a product term of light-intensity physical activity with age (≤67 versus >67 years of age at diagnosis), sex or chemotherapy (yes versus no). Statistically significant interactions (p-values<0.05) are denoted with an asterisk (*).  ^c^ The beta-coefficients represent the overall longitudinal difference in the outcome score per 8 hours∙week^-1^ difference in light-intensity physical activity, including intra- and inter-individual associations. Higher outcome scores indicate better global quality of life, physical, role and social functioning and more fatigue.  ^d^ The beta-coefficients represent the change in the outcome score over time within individuals per 8 hours∙week^-1^ increase in light-intensity physical activity.  ^e^ The beta-coefficients represent the difference in the outcome score between individuals, per 8 hours∙week^-1^ difference in light-intensity physical activity. | | | | | | | | | |

| **Supplemental Table 3.** Analysis of interactions by time since end of treatment of associations of light-intensity physical activity (per 8 hours∙week^-1^) on quality of life, functioning and fatigue in colorectal cancer survivors, including time modelled as a continuous variable (to explore linear interaction) as well as for separate time points (to explore non-linear interaction)^a^. | | | | |
| --- | --- | --- | --- | --- |
|  |  | β | 95% CI | P_interaction_^b^ |
| Global quality of life (0-100) | LPA (reference: 6 weeks)^c^ | 0.90 | -0.25; 2.06 | 0.127 |
|  | LPA*time(6 months)^d^ | 0.21 | -1.16; 1.57 | 0.766 |
|  | LPA*time(12 months)^e^ | 0.68 | -0.71; 2.07 | 0.342 |
|  | LPA*time(24 months)^f^ | 1.27 | -0.37; 2.92 | 0.130 |
|  | LPA*time (continuous: per month)^g^ | 0.05 | -0.02; 0.12 | 0.170 |
| Physical functioning (0-100) | LPA (reference: 6 weeks)^c^ | 1.05 | 0.17; 1.94 | 0.019 |
|  | LPA*time(6 months)^d^ | 0.04 | -0.96; 1.05 | 0.934 |
|  | LPA*time(12 months)^e^ | -0.03 | -1.06; 0.99 | 0.947 |
|  | LPA*time(24 months)^f^ | 0.02 | -1.19; 1.22 | 0.975 |
|  | LPA*time (continuous: per month)^g^ | -0.01 | -0.07; 0.04 | 0.637 |
| Role functioning  (0-100) | LPA (reference: 6 weeks)^c^ | 1.97 | 0.17; 3.77 | 0.032 |
|  | LPA*time(6 months)^d^ | -0.36 | -2.45; 1.73 | 0.736 |
|  | LPA*time(12 months)^e^ | -0.93 | -3.07; 1.21 | 0.394 |
|  | LPA*time(24 months)^f^ | 0.93 | -1.59; 3.45 | 0.471 |
|  | LPA*time (continuous: per month)^g^ | 0.00 | -0.11: 0.11 | 0.981 |
| Social functioning  (0-100) | LPA (reference: 6 weeks)^c^ | 0.64 | -0.66; 1.95 | 0.334 |
|  | LPA*time(6 months)^d^ | 0.53 | -1.03; 2.09 | 0.506 |
|  | LPA*time(12 months)^e^ | 0.52 | -1.07; 2.11 | 0.523 |
|  | LPA*time(24 months)^f^ | 0.84 | -1.04; 2.72 | 0.382 |
|  | LPA*time (continuous: per month)^g^ | 0.01 | -0.08; 0.09 | 0.861 |
| Fatigue (20-140) | LPA (reference: 6 weeks)^c^ | -0.67 | -2.09; 0.76 | 0.357 |
|  | LPA*time(6 months)^d^ | 0.29 | -1.35; 1.92 | 0.731 |
|  | LPA*time(12 months)^e^ | -1.29 | -2.96; 0.38 | 0.129 |
|  | LPA*time(24 months)^f^ | -2.19 | -4.16; -2.22 | 0.029* |
|  | LPA*time (continuous: per month)^g^ | -0.11 | -0.19; -0.02 | 0.016* |

Abbreviations: LPA, light-intensity physical activity; β, beta-coefficient; CI, confidence interval.

^a^ Models were adjusted for sex (male/female), age (years) and time since the end of treatment (months), chemotherapy (yes/no), number of comorbidities (0, 1 or ≥2), body mass index (kg∙m^-^²), cancer stage (I, II or III) and self-reported moderate-to-vigorous intensity physical activity (hours∙week^-1^). For role functioning, additional adjustment was performed for light-intensity physical activity at diagnosis (hours∙week^-1^) (see Methods).

^b^ Statistical interaction was tested by including a product term of the light-intensity physical activity variable with time in indicator variables (6, 12 or 24 months, with 6 weeks as reference) or modelled as a continuous variable (months). Statistically significant interactions (p-values<0.05) are denoted with an asterisk (*).

^c^ The beta-coefficients represent the overall longitudinal difference in the outcome score at 6 weeks, per 8 hours∙week^-1^ difference in light-intensity physical activity. Higher outcome scores indicate better global quality of life, physical, role and social functioning and more fatigue.

^d^ The beta-coefficients represent the difference between the overall longitudinal difference in the outcome score at 6 months compared to 6 weeks, per 8 hours∙week^-1^ difference in light-intensity physical activity.

^e^ The beta-coefficients represent the difference between the overall longitudinal difference in the outcome score at 12 months compared to 6 weeks, per 8 hours∙week^-1^ difference in light-intensity physical activity.

^f^ The beta-coefficients represent the difference between the overall longitudinal difference in the outcome score at 24 months compared to 6 weeks, per 8 hours∙week^-1^ difference in light-intensity physical activity.

^g^ The beta-coefficients represent the overall longitudinal difference in the outcome score per 8 hours∙week^-1^ difference in light-intensity physical activity, per 1 month increase in time since the end of treatment.

| **Supplemental Table 4**. Results from the time-lag analysis of longitudinal associations between light-intensity physical activity (per 8 hours∙week^-1^) and quality of life, functioning and fatigue at the subsequent time point in colorectal cancer survivors. | | |
| --- | --- | --- |
|  | β^a^ | 95% CI |
| Global quality of life (0-100)^b^ | -0.19 | -1.24; 0.86 |
| Physical functioning (0-100)^b^ | -0.09 | -0.78; 0.61 |
| Role functioning (0-100)^b^ | 0.75 | -0.77; 2.28 |
| Social functioning (0-100)^b^ | 0.57 | -0.84; 1.62 |
| Fatigue (20-240)^b^ | -0.81 | -2.11; 0.50 |

Abbreviations: β, beta-coefficient; CI, confidence interval.

^a^ The beta-coefficients represent the overall longitudinal difference in the outcome score at a later time point per 8 hour∙week^-1^ difference in light-intensity physical activity at the previous time point, including both intra- and inter-individual associations. Higher outcome scores indicate better global quality of life, physical, role and social functioning and more fatigue.

^b^ Fully adjusted models including adjustment for sex (male/female), age (years) and time since the end of treatment (months), chemotherapy (yes/no), number of comorbidities (0, 1 or ≥2), body mass index (kg∙m^-^²), cancer stage (I, II or III) and self-reported moderate-to-vigorous intensity physical activity (hours∙week^-1^). For role functioning, additional adjustment was performed for light-intensity physical activity at diagnosis (hours∙week^-1^) (see Methods).
